# Quality Is in the Eye of the Beholder—A Focus Group Study from the Perspective of Ambulance Clinicians, Physicians, and Managers

**DOI:** 10.3390/healthcare7010041

**Published:** 2019-03-13

**Authors:** Andreas Rantala, Lina Behm, Helena Rosén

**Affiliations:** 1 Department of Health Sciences, Lund University, SE-221 00 Lund, Sweden; lina.behm@hkr.se (L.B.); helena.rosen@med.lu.se (H.R.); 2 Emergency Department, Helsingborg General Hospital, SE-205 01 Helsingborg, Sweden; 3 Centre of Interprofessional Cooperation within Emergency Care (CICE), Linneaus University, SE-251 95 Växjö, Sweden; 4 Department of Health Sciences, Kristianstad University, SE-291 88 Kristianstad, Sweden

**Keywords:** quality, ambulance services, prehospital emergency care, focus group, ambulance clinicians, emergency medical services

## Abstract

Quality within all areas of healthcare should be systemically monitored and ensured. However, the definition of quality is complex and diverse. In the ambulance service (AS), quality has traditionally been defined as response time, but this measurement eliminates the possibility of addressing other characteristics of quality, such as the care provided. This study aimed to explore what constitutes quality in the context of the ambulance service as experienced by ambulance clinicians, physicians, and managers. A focus group study was conducted with 18 participants. The three focus groups were analyzed with the focus group method developed by Kreuger and Casey. The participants highlighted patient involvement, information and care, as well as adherence to policies, regulations, and their own standards as representing quality in the AS. This study demonstrates that quality is in the eye of the beholder. As quality seems to be viewed similarly by patients and ambulance clinicians, physicians, and managers, stakeholders should aim for a paradigm shift where patients’ experience of the care is just as important as various time measures.

## 1. Introduction

Quality in the Swedish ambulance service (AS) is traditionally defined as response time, which means the time from the patient’s phone call to the emergency medical dispatch centre to the arrival of the ambulance at the scene [1]. However, measurement of response time eliminates the possibility of addressing other characteristics of quality, such as the care provided. Therefore, the rationale behind this study is to explore aspects of quality in the Swedish AS from the perspective of ambulance clincians, physicians, and managers.

In common with almost all healthcare in Sweden, the AS is organized by 21 county councils, which creates variation in structure and operation throughout the nation. Nationally, ambulance clinicians (ACs) within the Swedish AS consist of at least one registered nurse (RN) and one emergency medical technician (EMT). However, some county councils have decided to crew their ambulances with at least one specialist ambulance nurse (SAN) together with an RN or EMT [2,3]. Physicians within the AS in Sweden mainly operate in the helicopter emergency medical services (HEMS) and rarely in land-based ambulances. However, senior ambulance physicians in Swedish AS organizations are traditionally responsible for the production of guidelines and standing orders for RNs and SANs, dealing with medical deviation reports and educational tasks [4]. In the Swedish healthcare system, quality is regulated through legislation issued by the Swedish National Board of Health and Welfare [5], which is compulsory for managers in general and operational managers in particular. In addition, the county councils often issue supplementary quality indicators, such as response time. However, within the AC team the RN or, if applicable, the SAN, is responsible for the operational quality of care [6,7]. The need to define, identify, communicate, measure and evaluate the quality of care has increased in line with the professionalization and academization of the AS, where RNs hold a bachelor degree in nursing. The advanced level SAN education leads to a Postgraduate Diploma in Specialist Nursing Pre-hospital Emergency Care and a Master of Science Degree (60 credits) in Nursing/Caring Science at most universities [8,9]. Quality development is also described as one of the six core competencies (person-centred care, teamwork and collaboration, evidence-based practice, quality development, safety and informatics) for healthcare professionals, which includes nurses [10,11].

The definition of quality of care is complex and diverse. Donabedian [12] highlights the fact that the concept of quality of care has a dual meaning; one part is of a technical nature, covering the structure, processes, and results of care, while the second concerns the experience of quality of care, defined as what care recipients can value. This has been further developed by, for example, Wilde, et al. [13], who describe quality of care as an overall concept based on norms, expectations, and experiences that patients bring with them when encountering the healthcare system and its staff. Similarly, in view of the fact that the Swedish AS is staffed by RNs, quality can be seen in the light of nursing-sensitive outcome indicators (NSOI). These are described by the American Nurses’ Association as “how patients and their conditions, are affected by their interaction with nursing staff” [14]. NSOI may help to shed light on the nursing service setting and encourage nurses to undertake quality monitoring and reporting [15]. Almost [16] states that NSOI can be classified into four groups: Functional, which comprises physical and psychosocial functioning in addition to self-care capacities; Clinical, which comprises management and symptom control; Perceptual, which involves satisfaction with nursing care and the care outcome; and Safety, which includes adverse events and complications. Research shows that the perspectives of society (patients and significant others) and healthcare professionals differ to some extent. Healthcare professionals often work according to guidelines for safe care, which involve measuring, streamlining and ensuring the quality of care, as well as providing adequate information. On the other hand, patients highlight the need to tell their story, report the problems they perceive, be seen as individuals and be invited to participate in the care on their own terms, as a basis for good healthcare [17,18,19]. The quality of care has been sparsely described from an ambulance context viewpoint. A UK-based consensus study highlighted clinical patient outcomes, for example, discharged alive from the hospital, reduction in pain after administered analgesia and re-contacting the AS within 24 h, as well as response time and correctly performed assessments/triage as potential quality measurements in an AS setting [20]. There are also examples in specific areas within the AS context, such as effectiveness of chest compressions [21] and cardiac arrest survival [22].

On the basis of theories, research and the fact that the Swedish Health Care Act [23] requires that quality within all areas of healthcare should be systematically and continuously monitored and safeguarded, there is a need to elaborate on the quality of care perspective of the healthcare professionals within the Swedish AS.

### Aim

The aim of this study was to explore what constitutes quality in the context of the ambulance service as experienced by ambulance clinicians, physicians and managers.

## 2. Materials and Methods

### 2.1. Design

The focus group method developed by Kreuger and Casey [24] was used in this study to explore the participants’ experiences of what constitute quality in the AS context. In focus group studies, data are generated by discussion among the participants and the interaction during the focus group sessions is a part of the method [24].

### 2.2. Sample and Participants

All the participants were recruited from or in connection with a workshop hosted by the AS in the south of Sweden in 2015. The aim of this workshop was to gather knowledge to develop a national quality register in the AS. Present and former AS clincians, physicians, and managers as well as teachers and researchers in the field all over Sweden were invited to attend the workshop. The ambition was to obtain a broad range of knowledge and experience from different professionals, age groups and gender. The invitation was sent by e-mail and included information about the focus groups. Prior to the workshop, one pilot interview was conducted to test the interview questions. The participants in that focus group (*n* = 5) were recruited from those who received invitations but could not take part in the workshop. The pilot interview was included in the analysis as the researchers judged that the interview guide functioned well. At the workshop, the participants were again informed about and asked to participate in the focus group study in order to obtain their view of what constitutes quality in the AS context. The participants were divided into groups with the aim of obtaining a spread in gender, age and profession to maximise the opportunity to explore different experiences within each group. A total of 18 persons from several counties in Sweden were interviewed in three focus groups. The study participants ranged in age from 32–60 years (mean age 46 years) and the three focus groups contained both younger and older participants, see Table 1. Of the participants, 44% were female, 83% were SANs and 17% were physicians. Their work experience differed from three to 28 years with a mean experience of 11 years. Two of the physicians served as operational managers (males, aged 45 years with five years of experience as a manager and 60 years with 20 years of experience as a manager). One of the SANs was a unit manager (male, aged 39 years with 11 years of experience as a manager).

### 2.3. Data Collection

All the focus groups included one moderator (Lina Behm or Helena Rosén) who led the focus groups and a secretary who took notes and observed the interaction in the focus groups. The moderators had previous experience of conducting focus group sessions but no experience of the AS context. The researchers selected a neutral setting for the focus groups that was new for all the participants. A guide for the focus group sessions with one main question; What constitutes quality in the AS context? Follow-up questions were employed. Probing questions which differed according to the responses and discussions among the participants were used to deepen the discussion. The moderator made a great effort to make sure that all participants were active and received an opportunity to express their views during the focus groups. The three focus group sessions lasted for an average of 127 min (range 115–142) and were recorded and transcribed verbatim.

### 2.4. Data Analysis

The analysis of the data was conducted according to the focus group method developed by Krueger and Casey [24], which focuses on analysing the discussions between the participants rather than individual opinions. Hence, the focus group discussions were analysed like a conversation and not like an interview. According to the method, the analysis followed the steps described below. The first step started during the focus group sessions when the moderator (HR and LB) attempted to capture the essence of the discussion and the entire analysis process was guided by the aim of the study. After the focus group sessions, one of the researchers (HR) read the transcribed discussions and listened to the recordings several times to gain an impression of the data. Discussions relevant to the aim of the study were identified in the raw data and sorted into categories. In order to deepen the understanding, the next step was to summarize and interpret the categorized data. The co-author LB also read the transcribed discussions to extract preliminary codes and categories. An independent analysis was conducted by the researcher (LB) and after that, both researchers (HR and LB) compared and discussed their analysis in several meetings. The reviews of the similarities and differences between the codes and the preliminary categories led to the development of themes and sub-themes. Finally, all of the authors reviewed the findings until consensus was achieved. One theme, two categories, and eight sub-categories emerged. In the final step quotations (discussions) that illustrated the sub-categories were selected from the interviews. The consolidated criteria for reporting qualitative research (COREQ) checklist was used to ensure that important aspects of the study were reported [25].

### 2.5. Ethical Considerations

Ethical considerations regarding consent, confidentiality, utility, and information was safeguarded and are in line with those of the Declaration of Helsinki [26]. Verbal and written information about the study was provided and all participants gave their written informed consent. The study did not involve patients, which means that ethical approval was not required in accordance with the Swedish Law concerning Ethical Review or Research Involving Humans (SFS 2003:460).

## 3. Results

Overall, quality in the AS was perceived as difficult to define as the mission statement of the Swedish AS was somewhat unclear to the participants. There were differing opinions about whether healthcare professionals or patients determine what constitutes quality in the AS. However, there was consensus was ‘*Quality is in the eye of the beholder*’. It was clear that ambulance clinicians, physicians and managers, from here referred to as a participants, believed that quality could mean different things to healthcare professionals, the public and the organization. The participants shared what they experienced as constitution quality from their different perspectives. The theme ‘*Quality is in the eye of the beholder*’ can be explained by two categories; *Patients appreciate the care provided*’ and ‘*ACs’ adherence to policies, regulations*, *and their own standards*’.

### 3.1. Patients Appreciate the Care Provided

It was expressed that when assessing what characterizes quality, different quality parameters such as patients’ experiences of the care must be taken into account, not just statistics, such as response time. The main category, ‘*Patient appreciate the care provided*’, reveals the participants different experiences of what constitutes an appreciative patient and is explained by five subcategories (Table 2). The impressions was that patients appreciated the care: ‘*When the patient and significant others feel involved*’; ‘*When the patient feels that she/he has been informed*’; ‘*When the patient feels healthier*’; ‘*When the patient feels that she/he has been cared for in an appropriate manner*’; and ‘*When the patient experiences a sense of security*’.

#### 3.1.1. When the Patient and Significant Others Feel Involved

The participants believed that a prerequisite for patient to appreciate the care is that she/he and significant others feel involved. Involvement in the care was explained by the participants as the patient being seen, heard, listened to, and confirmed as a person. Different views on how patients could be involved in the care were mentioned by the participants, for example, being able to choose between whether to stay at home with self-care advice or be transported to the accident and emergency department (AED) by ambulance. Nevertheless, a certain consensus emerged that the sense of involvement exists when patients feel that their personal experiences and knowledge of their illness are taken into consideration. However, the participants also pointed out that not everyone wants to be involved in their own care.

When I talk about participation, I am not talking about choosing different treatment methods. I mean that we take the patient’s previous experiences and knowledge of his own illness and such things into consideration.

(Informant 15)

But we already do that and measure it, is that not nursing? Well, it is.

(Informant 16)

#### 3.1.2. When the Patient Feels That She/He Has Been Informed

Information about the patient’s condition, why a treatment was initiated as well as explaining to the patient why she/he is left at home was seen as quality. The participants believed that when patients are informed about the course of events they feel more content.

We have so much more knowledge, we know when an ECG needs to be performed. We know that we may need to send this ECG for someone to make an assessment and such things. While for the patient, transport to hospital as soon as possibly means quality. So there we also have different opinions on what quality is/on what constitutes quality.

(Informant 15)

#### 3.1.3. When the Patient Feels That She/He Has Been Well Cared for/Has Been Cared for in an Appropriate Manner

This sub-category reflects the participants’ experience that quality within the AS means that the patient is content with how she/he is treated by the ACs. The participants believed that patients’ experience of the AS is dependent on the quality of their encounter with the ACs and that they are appreciative when listened to and taken seriously. Experiences of difficulties ensuring that certain patient groups were content with the AS encounter were mentioned, for example, patients with an addiction. The participants believed that for patients to feel well cared for in an appropriate manner, ACs must be flexible and ask what is important to the patient.

As a boss I sometimes get asked why did you do that, is it allowed? But if it is right for the patient yes, it’s the right thing to do. I think sometimes we forget why we go to work because that is where quality arises. In our business quality only occurs in the meeting between two people. I cannot say that it occurs elsewhere. The other aspects are so obvious, for example, that the vehicles work correctly, that the air pressure is correct, that is not quality. Quality is in the encounter.

(Informant 6)

60–70% of our job involves encountering other people. It does not matter whether you are a physician or an ambulance nurse, nurse or whatever, you need to connect with the other person.

(Informant 9)

#### 3.1.4. When the Patient Feels Healthier

According to the participants, when patients seek help from the AS they lack knowledge and are often worried and want to be cared for. In some cases, patients call the AS in a crisis that is perhaps the worst thing that ever happened to them. The participants believed that patients need reassurance that someone knows what care intervention is necessary. Thus, it was suggested that in this case good quality is when the patient experiences improvement of the perceived illness. The participants believed that the patients’ experience of receiving the treatment they needed was important for their subjective perception of illness.

My definition is that they should be a bit healthier when we are done. As simple as that. Experience of feeling healthy. It’s not always something we can do objectively, but the experience should be that after meeting healthcare professionals they feel a bit healthier.

(Informant 6)

#### 3.1.5. When the Patient Experiences a Sense of Security

Quality ambulance care was considered to emerge when the patient feels safe and dares to trust the ACs. The participant’s believed a feeling of safety to be something very basic but extremely important.

That they should dare to surrender themselves to our care. So, I think that the concept of safety is on the same “level” as the sheets on the ambulance stretcher being smooth, which is as natural and important as the patient feeling safe.

(Informant 16)

In order for patients to experience the AS care as safe, it had to be person-centred, satisfy all kinds of patient need, be of high quality and given in a timely manner. The participants felt that conveyance to the AED often resulted in greater safety for the patient compared to driving to the hospital in her/his own car.

Yes, you get there [to the hospital] faster, the time factor is an important aspect of quality.

(Informant 6)

#### 3.1.6. ACs’ Adherence to Policies, Regulations, and Their Own Standards

The participants experienced that quality ambulance care means adhering to laws, guidelines and regulations, but also to their own standards. The skills of ACs could vary, which had a bearing on how the patient’s care needs were addressed and documented. They were also dependent on the organization and/or AS culture. *ACs’ adherence to policies, regulations and own standards* is based on three sub-categories; ‘*When nursing and medical needs are met*’, ‘*When competence descriptions and internal regulations are followed*’, and ‘*When the use of technology is efficient*’ (Table 2).

#### 3.1.7. When Nursing and Medical Needs Are Met

The participants perceived that quality was associated with if and how basic needs were met and that the correct treatment in relation to the assumed diagnosis was given. The participants experienced that they had a good opportunity to spend time with the patient due to the fact that the AS could only take care of one patient at a time. This was considered of great importance for the provision of good quality care and made it possible to fully utilize their nursing skills. It was expressed that during conveyance by ambulance it was possible to fulfil both basic needs such as facilitating breathing and more comprehensive needs such as informing the patient about what was going to happen. In some cases the mandatory regulations and guidelines were considered to hinder the ACs’ ability to fulfil the patient’s nursing needs. However, the ability to make a correct assessment was found to be of the utmost importance.

Documentation clearly emerged as an important part of meeting nursing and medical needs. Adequate documentation of the assessments, measurements and evaluations performed was considered a quality indicator. However, the participants reported that the prerequisites for such documentation were lacking. Obstacles were lack of time and the restraints imposed by the electronic documentation system. The participants stated that being unable to follow-up care due to the absence of documentation is both frustrating and serious.

We have to document the encounter with the patient. But what should we document? Should we just document medical figures and facts or should we start documenting nursing? What nursing should we document? Has everyone been involved in nursing or... but what was it like in the home. Was the patient at risk?

(Informant 2)

#### 3.1.8. When Competence Descriptions and Internal Regulations Are Followed

Competence was highlighted as a quality indicator and assumed to depend on compliance with the competence descriptions for SANs and county council regulations. The fact that the regional requirements for further training and education varied between the different county councils throughout the country was described as a dilemma, which also influenced the regional AS culture. Hence, ACs from different county council organizations or AS cultures could not cooperate effectively.

The participants highlighted the importance of AS staff training for the ability to assess patients correctly. They believed it was vital for all nurses to have the same training, irrespective of their specialist nursing education and workplace location.

The quality concept becomes clear if you study your own actions or see the care that relatives or friends have received. For example, take a vital area like airway management. I would expect the organization to ensure the necessary competence for ventilating a relative or friend of mine. But I don’t expect that we should be able to intubate as an anaesthesia nurse does that well, which is fine. But in some cases we have to use a laryngeal mask. I would be very disappointed if an AC could not even lift the chin (to free the airways) that’s a basic skill.

(Informant 16)

The broad range of skills possessed by specialist nurses could represent quality in some situations. However, the participants felt that the main competence in the AS was that of the SAN.

*The range of specialist nursing careers in the AS currently varies a lot while ambulance care is a speciality in its own right (the specialist ambulance nurse/SAN*).

(Informant 14)

#### 3.1.9. When the Use of Technology Is Efficient

The participants perceived that quality involved following the rules and guidelines for the management and operation of the ambulance itself, but also for the medical equipment. They stated that ACs are extremely dependent on the technical systems in the ambulance, making it essential that the technology is used in an optimal manner. A dominant topic in deviation reporting and conversations between managers and ACs is technical problems with radios or the vehicle itself. The need for well-functioning technology was seen as something unique to the AS context.

Is it quality that it [technology] always works? Or is it quality to be contactable by radio? Is it achievable, or that the radio communication covers all geographic areas, or is it the sound quality? There are so many different aspects in radio communications alone.

(Informant 15)

Quality regarding the skill of driving the ambulance and handling the technical equipment was related to time measurements, for example, how long it takes the ACs to respond to assignments and the time from the patient’s phone call to the arrival of the ambulance at the scene. Competence was considered necessary in order to initiate proper treatment, described as familiarity with the equipment that sends an ECG to a physician who analyses it and determines the next step in the care chain. Quality was also perceived to involve aspects of radio communication such as handling the equipment correctly to ensure audibility and accessibility.

The call from the medical dispatch centre is measured. We actually have the ability to measure technology, as well as how quickly we respond to the assignment and are on our way in the ambulance and so on …

(Informant 16)

## 4. Discussion

This study reports ambulance clinicians’, physicians’, and managers’ experience of what constitutes quality within the AS context. In general, perceived quality in the healthcare system is not only an effect of the technical care provided, but includes interpersonal care relationships and the environment in which the care is delivered [27,28,29,30]. There is evidence indicating a direct link between a positive healthcare experience and improved patient safety, clinical efficiency and better health outcomes [31,32,33], as well as reduced healthcare costs and improved employee satisfaction [34]. The findings in this study highlight the fact that the participants recognize that quality is both patient appreciation with the care provided and adherence to policies, regulations, and their own standards. This duality is consistent with Donabedian’s description of quality as on the one hand acknowledging patients’ experience of quality of care and on the other quality indicators of a more technical and structural nature [12]. Quality was also perceived when ACs were able to spend time at the scene, which is highly valued by patients [35].

Previous studies indicate that interpersonal caring aspects in addition to medical treatment are important for patients treated by the AS [36,37,38]. A qualitative study of 20 patients in the AS concluded that the initial meeting is of vital importance for how patients experience pre-hospital care [39]. Research from ACs’ perspective demonstrates that the perceived main mission is to provide medically focused interventions. However, caring aspects are more or less explicitly expressed [40,41]. Our study reveals that the participants stressed the importance of involving the patients and significant others in the care, which is in line with person-centred care [42,43] that emphasizes listening and confirming the patient as a person. Person-centred care and patient satisfaction are considered important aspects of quality of care [43,44]. From the patient perspective, involvement in emergency care have been shown to concern being acknowledged as a competent person, not being forced to subordinate to the prevailing contextual culture and that ACs are attentive to the patient’s worries and aspects other than purely medical ones [45]. In this respect the study participants agree with previous research on patients in the AS setting [38] that highlights the importance of taking patients seriously and alleviating suffering. However, ACs tend to struggle with taking patients seriously, especially when they do not meet the ACs’ subjective criteria for emergency care [46]. According to the participants in the present study, involvement in the care could be enabled by inviting patients to participate in the decision-making process about whether or not they should be conveyed to the hospital or to other levels of care, for example, primary care facilities or remain at home with self-care advice. It has been shown that it is of importance that patients are involved in this process both in general [47] and in the specific context [35,38]. Despite the fact that previous studies reveal that ACs want to approach every patient equally, with respect and overcome possible preconceptions [46,48,49], patients presenting with an illness of low acuity were considered to be misusing ambulance resources, thus creating a conflict with the ACs’ wish to be available for higher priority assignments [49]. The possibility of ensuring quality is further complicated by the fact that ACs perceive a lack of support from the organization and struggle with formal directives relating to patients who are assessed as not in need of emergency ambulance care. This gives rise to the relatively new phenomenon of non-conveyance [46]. The participants agreed that if the documentation in patient records could not be performed properly, it would hinder the development of quality indicators. They also stated that being unable to follow-up care outcomes was frustrating.

Quality was also perceived in relation to different measurements such as time of response from the initial call to the emergency dispatch center to arrival at the scene. The preparedness to reach the patient as quickly as possible is in line with a study by Holmberg and Fagerberg [41], but utilization of lights and sirens is not entirely positive [50]. While lights and sirens improve the response and transport time, their use has no clinical effect on patient outcomes. Furthermore, as lights and sirens have unfavourable effects on patient safety, ACs and the general public, it is recommended that a protocol should be developed to minimize their usage [50]. Another quality indicator related to response time is cardiac arrest survival [22]

In the present study, following guidelines was shown to be important, which is supported by national and international studies [51,52,53] and recognized as a means of improving quality [54]. However, the participants experienced that guidelines and protocols mainly had a medical focus, making them potential obstacles to being open to patient needs [55]. It has been found that assessments that focus on the patient’s medical condition instead of her/his lifeworld, situation, and suffering can be an obstacle to fully understanding the individual and thereby her/his illness [56]. Thus guidelines should be revised to include the patient perspective as an essential part of the assessment.

The participants valued competence and knowledge, which they considered important quality indicators. However, the literature is not consistent in terms of what constitutes competence, which includes the AS context [57,58,59]. For example, an expert panel identified 44 separate competencies in ten areas, for example leadership, communication, professional and technical skills [58]. In contrast, AS managers ranked knowledge to assess the patient’s condition from a holistic viewpoint as the most important competence, followed by medical understanding to assess and care for diverse diseases as well as knowledge to care for critically ill patients [60]. Another study [61] highlighted the ability to manage the dialogue with the patient and be flexible, open and avoid being governed by predetermined statements. These competences are in line with the result of our study that highlights interpersonal caring aspects as quality of care. It also emphasized that the varied education and training in the country resulted in uneven quality in the practice of the profession.

Trustworthiness was safeguarded throughout the study. To enhance credibility [62] and to enrich the variation in the phenomenon under study, we aimed to recruit female and male participants with different professions and a range of work experience (in years) from various AS organizations. Focus groups made up of participants who do not know each other have been shown to facilitate interaction [63] which was the reason for decision. The variation as well as the number of participants in each group was successful as the discussions functioned well. This could also be a result of the work by the moderator and co-moderator to ensure that all participants played an active part in the discussions. One limitation was that the groups did not include an EMT and in one group there was no physician which might have had affected the interaction in either a positive or a negative way. However, we believe that the interaction functioned well in all groups. Another limitation was that the participants reported their view of what constituted quality from a patient perspective. This must be taken into consideration when interpreting our results. The participants were all clinically active and some had a leading role, which is a strength of the study. Studies about what constitutes quality from the patient’s perspective are recommended.

Furthermore, to ensure that the findings are credible, all authors were involved in the data collection and analysis. The analysis was first conducted by two of the authors (LB and HR), while the other author (AR) later verified the analysis, which strengthens credibility [64]. The theme and the categories were seen in all the focus groups and throughout the interviews the participants confirmed each other’s statements. The results are illustrated by quotations, which is a way to enhance the conformability of the study [65]. To facilitate transferability [62], the study provides a detailed description of participant characteristics, the data collection, and analysis process.

With regard to dependability [62], a guide to address the research question was specially developed and tested in a pilot study. The guide provided the moderator with a tool to keep on track and cover topics of interest in relation to the aim. The two authors who conducted the analysis had no experience of the AS, which increased their ability to remain objective during the analysis and also enhances conformability [65].

## 5. Conclusions

According to ambulance clinicians, physicians, and managers in Sweden, quality is in the eye of the beholder. There is a dualistic viewpoint that highlights both the patient-AC relationship and adherence to medical needs, guidelines, and technological skills.

Similar to previous studies of patients’ experiences from other healthcare settings, this study adds valuable knowledge that quality is viewed in the same way by patients and ACs, physicians and managers. This means that stakeholders should aim for a paradigm shift, where patients’ experience of the care provided is equally important as for example different time measurements. The possibility of following-up the care in patient records as part of quality assurance should also be increased. It is of the utmost importance that a high level of professional knowledge is created nationally and that it is possible to effectively measure the care provided. This is a prerequisite for the production and design of quality indicators to ensure quality care.

## Figures and Tables

**Table 1:** Demographic data of the participants in the study, *n* = 18.

Variables	Total (*n* = 18)	Focus Group 1 (*n* = 5)	Focus Group 2 (*n* = 6)	Focus Group 3 (*n* = 7)
Gender, *n* (%)				
Female	8 (44)	3 (60)	2 (33)	3 (43)
Age, median (range)	46 (32–60)	33 (32–52)	48 (42–60)	46 (36–53)
Profession, *n* (%)				
Specialist Ambulance Nurse	14 (77)	5 (100)	3 (50)	6 (86)
Physician	1 (6)	0 (0)	1 (17)	0 (0)
Manager (physician)	2 (11)	0 (0)	1 (17)	1 (14)
Manager (SAN)	1 (6)	0 (0)	1 (17)	0 (0)
Work experience in years, median (range)	11 (3–28)	8 (3–16)	15 (5–28)	11 (3–26)

**Table 2:** Summary of the theme, categories and sub-categories that describe how ambulance clinicians, physicians, and managers experience what constitutes quality in the AS context.

Quality Is in the Eye of the Beholder	
Patients appreciate the care provided (Based on the participants’ impression that patients appreciated the care:)	ACs’ adherence to policies, regulations and their own standards
When the patient and significant others feel involved	When nursing and medical needs are met
When the patient feels that she/he has been informed	When competence descriptions and internal regulations are followed
When the patient feels that she/he has been cared for in an appropriate manner	When the use of technology is efficient
When the patient feels healthier	
When the patient experiences a sense of security	
